# A Survey of the Thermal Analysis of Implanted Antennas for Wireless Biomedical Devices

**DOI:** 10.3390/mi14101894

**Published:** 2023-09-30

**Authors:** Ala Alemaryeen, Sima Noghanian

**Affiliations:** 1 Department of Computer Engineering and Communication, Tafila Technical University, Tafila 66110, Jordan; 2 CommScope Ruckus Networks, Sunnyvale, CA 94089, USA; sima_noghanian@ieee.org

**Keywords:** antenna, bioheat transfer, electromagnetic heating, finite element method (FEM), safety

## Abstract

Wireless implantable biomedical devices (IBDs) are emerging technologies used to enhance patient treatment and monitoring. The performance of wireless IBDs mainly relies on their antennas. Concerns have emerged regarding the potential of wireless IBDs to unintentionally cause tissue heating, leading to potential harm to surrounding tissue. The previous literature examined temperature estimations and specific absorption rates (SAR) related to IBDs, mainly within the context of thermal therapy applications. Often, these studies consider system parameters such as frequency, input power, and treatment duration without isolating their individual impacts. This paper provides an extensive literature review, focusing on key antenna design parameters affecting heat distribution in IBDs. These parameters encompass antenna design, treatment settings, testing conditions, and thermal modeling. The research highlights that input power has the most significant impact on localized temperature, with operating frequency ranked as the second most influential factor. While emphasizing the importance of understanding tissue heating and optimizing antennas for improved power transfer, these studies also illuminate existing knowledge gaps. Excessive tissue heat can lead to harmful effects such as vaporization, carbonization, and irreversible tissue changes. To ensure patient safety and reduce expenses linked to clinical trials, employing simulation-driven approaches for IBD antenna design and optimization is essential.

## 1. Introduction

**Figure 1:**
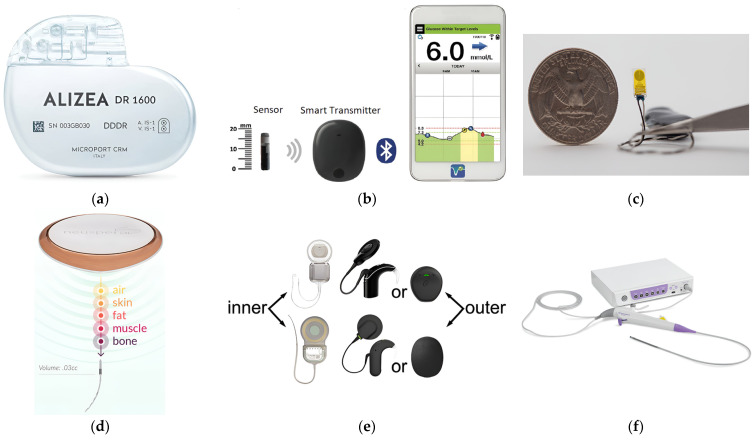
Examples of available commercial IBDs: (**a**) Pacemaker [20]; (**b**) blood–glucose sensor [21]; (**c**) temperature sensor [22]; (**d**) neurostimulation therapy implant [23]; (**e**) hearing aids [24]; and (**f**) imaging device [25].

Wireless implanted biomedical devices (IBDs) are highlighted as essential components of the modern-day healthcare industry. They are a promising modality for various diagnostic [1,2] and therapeutic [3] clinical applications. Such applications include pacemakers [4], blood–glucose sensors [5], temperature monitoring [6], retinal implants [7], and imaging devices [8]. Examples of available commercial IBDs for different medical purposes are shown in Figure 1. In addition, IBDs are widely used for thermal therapies such as hyperthermia and microwave ablation (MWA) for tumor treatment in the lung [9], liver [10], breast [11], bone [12], prostate [13], and kidney [14]. Those therapies offer various advantages over traditional treatments, including (1) minimal invasiveness: involving incisions of approximately 3 mm, hence the recovery process can be accelerated; (2) effective heating: breaking the hydrogen bonds within the natural structure of proteins, leading to the impairment their functionality and causing cellular destruction [15]; and (3) ease of functionality: particularly for patients who are not surgical removal candidates due to limitations such as multiple tumor sites, tumor located in proximity to critical blood vessels, and tumor size [16]. In the case of hyperthermia treatment, the temperature is maintained within the range of 41 °C to 45 °C, while in the case of MWA treatment, the temperature exceeds 60 °C. Despite these potential benefits of IBDs, it is important to note that the constant communication between the human body and the external world is via electromagnetic (EM) radiation using an antenna. EM fields interacting with the human body could result in power dissipation within healthy tissues and, in turn, an undesired increase in temperature. In healthy tissue, for every 1 °C rise beyond 37 °C, the basal metabolic rate increases by approximately 10–12%. As a result, the heart rate accelerates, and there is an augmented need for water and calories [17]. Healthy tissues when exposed to EM radiation are at risk of various health hazards, including brain tumors, childhood leukemia, and the escape of albumin across the blood–brain barrier [18]. Furthermore, the electric conduction via IBDs can lead to tissue burns, affecting the surrounding tissues of both active and passive IBDs. The greatest risk is generally associated with small exposed regions that are in direct contact with the device [19]. Electrosurgery serves as an example of IBDs that is often associated with reports of adverse incidents and was included in the Emergency Care Research Institute’s (ECRI) report as one of the top 10 patient-related safety events in 2012. Sparks generated during electrosurgery have the potential to cause surgical fires occurring within the operating room, with the risk elevated when the tissue is saturated with high levels of oxygen or when combined with flammable substances such as alcohol [19].

The concern regarding the potential adverse effects of EM radiation on human healthy tissues has been extensively debated for over a decade. To estimate the level of EM absorption by a unit mass of the human tissue, a metric known as specific absorption rate (SAR) is utilized. Institutions such as ICNIRP and IEEE [26,27] formulated guidelines that rely on SAR values to regulate the interaction with EM radiation. However, the absorption of EM radiation in tissues leads to an increase in temperature, which is recognized as the primary cause of biological hazards in healthy tissues. Studies have emphasized the significance of studying SAR [28], as well as temperature elevation [19] in healthy tissues when exposed to EM radiations [29]. It is worth mentioning that most of the previous studies primarily focused on SAR optimization while using IBDs and often neglected the consideration of heat transfer and ensuring the absence of hot spots within the targeted biological tissue [26], leading to an incomplete assessment of the study outcomes. Moreover, the safety of the implanted antenna relies more on the temperature change induced within the tissue rather than the actual power density value [30,31]. In the study reported in [19], using an in vitro model, the tissue temperature adjacent to metal implants during electro-surgery was estimated to have a thermal coefficient of 0.088 °C/W/min. At a power level of 60 W, the temperature could rise to over 43 °C in just 1 min. Consequently, there is a notable risk of elevated tissue temperature when using extended activation durations or high-power values, which increases the potential risk of patient injury. Therefore, there is a need for heat transport modeling to comprehensively explain the actual process of IBD activation inside the human body. 

The major method to reduce tissue heating (i.e., including SAR) is to reduce the input power. However, there are some other works around, such as the structure and placement of the implanted antenna. There might be some occasions where there is a choice of placing the implant in different tissue materials to reduce the heating and SAR. In most cases, there is no such option. Another factor to control is the gain and radiation patterns. One may use an antenna with an omnidirectional or as close as possible to an isotropic pattern to distribute the power around the tissue and not cause any hot spots. Some papers reported the use of ground plane or periodic structures such as electronic band gap structure to minimize the radiation toward the inner part of the body and increase the gain toward the outside; therefore, a reduced power combined with the higher gain can be used to reduce SAR. The other option is to use a layer of insolating heat-absorbing material around the antenna to insulate the antenna from the tissue. Reducing the discontinuities of the near E field using a Ferrite sheet and the use of metasurface techniques were also proposed in the literature to reduce SAR levels [28,29,30,31,32]. 

Many parameters influence the thermal rise observed during the IBD’s operation. Moreover, each individual possesses distinct biological characteristics, including tissue density, mechanical and electrical properties, and physiological characteristics [33]. Consequently, there is a significant need to conduct parametric studies to evaluate the impact of different parameters on the heat dissipation of IBDs and, subsequently, their overall efficiency [34]. Numerical modeling plays a crucial role in conducting parametric studies, as it allows for assessing the significance of underlying phenomena and facilitates improved optimization approaches of new IBD designs. Hence, it serves as a fast, convenient, and cost-effective means of evaluating and refining the device during the prototyping phase. In addition, numerical modeling offers a highly fast and controlled environment for simulating and evaluating device performance. A prime example is the clinical treatment involving MWA and hyperthermia procedures, where precise temperature control is necessary to ensure the selective elimination of cancer cells while minimizing harm to healthy biological tissues. Hence, both numerical and experimental models have been extensively employed as informative tools to investigate IBD activation within biological tissues. These models play a crucial role in offering essential treatment planning information to clinical practitioners for practical treatment strategies. They assist in determining the optimal placement of the device and the appropriate amount of microwave power to be delivered via the antenna. In addition, in the case of thermal therapy treatment procedures, by predicting the transient temperature profiles and the extent of damage within the target tissue while minimizing harm to the surrounding healthy tissue, these models define the duration of irradiation necessary for achieving complete tumor necrosis [35]; thus, they will enable precise and optimized treatment outcome.

It is worth mentioning that different IBDs can be used to target different biological tissues of different compositions, shapes, sizes, and electrical and thermal properties. Thus, it is important to investigate how various factors affect the heat dissipation of IBDs within the biological tissue and, in turn, consider the human body’s safety. The objective of this study is to identify the parameters that are responsible for electromagnetic heating and the parameters that influence the transfer of bioheat energy. This will improve our understanding of the impacts of IBDs’ structure and design on the heat distribution in the surrounding biological tissues.

## 2. Methodology

**Figure 2:**
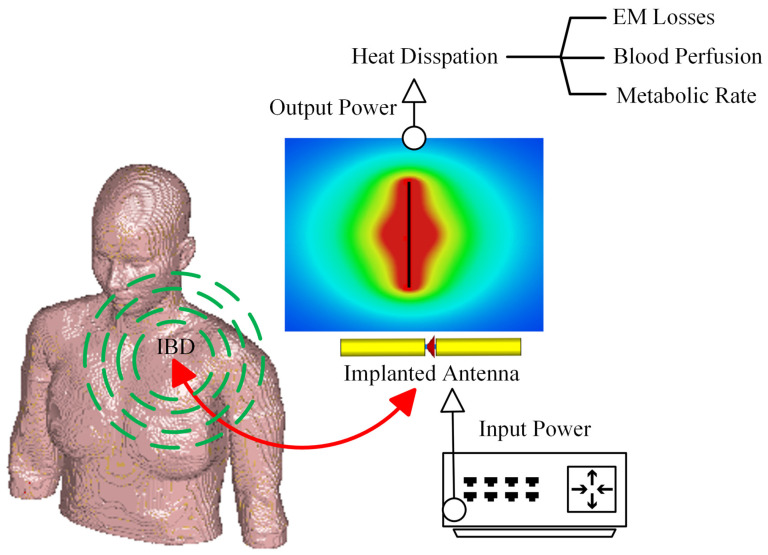
A schematic of the wireless IBD thermal studies.

**Figure 3:**
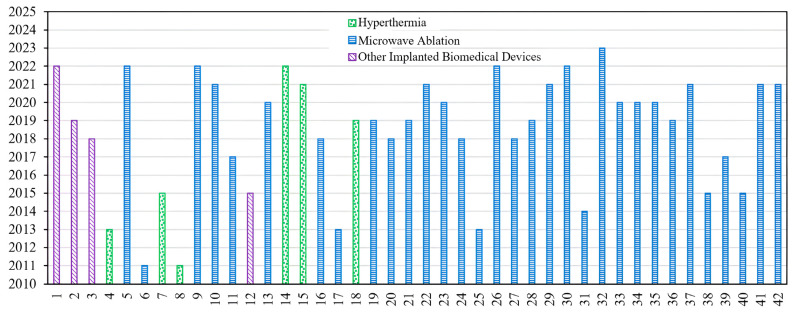
Considered thermo-electric studies that were reported in the literature. Numbers are linked to references listed in Table 1.

**Figure 4:**
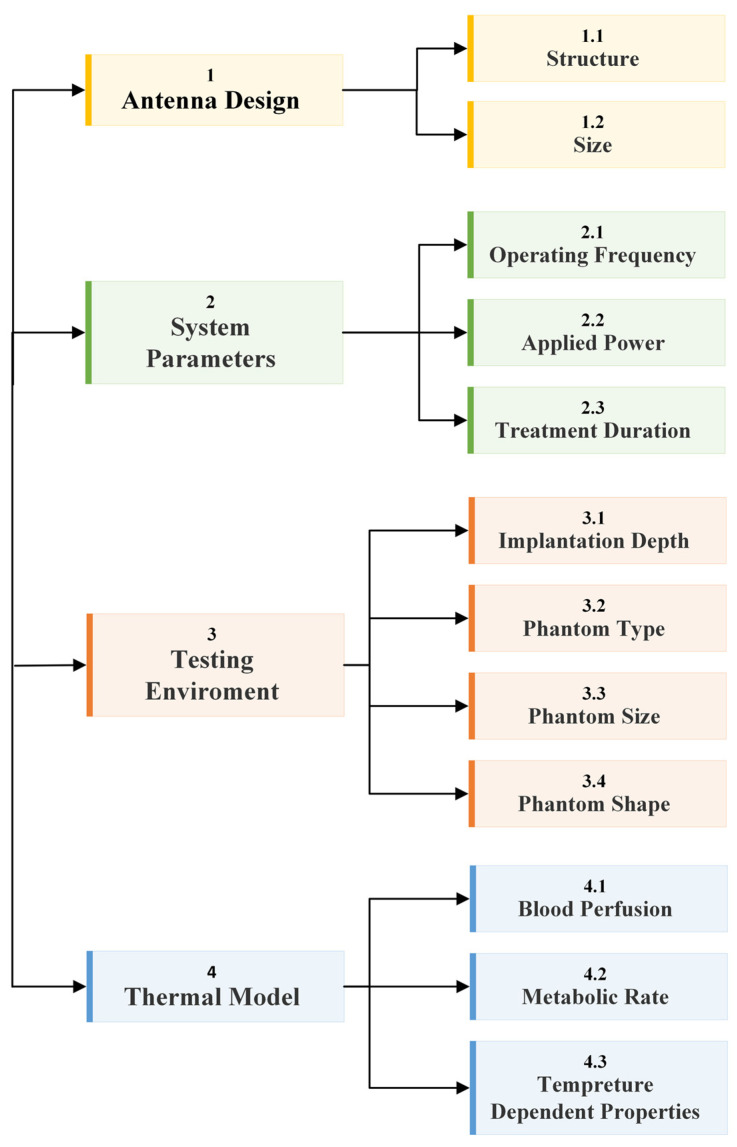
A diagram of this paper’s structure and how the studied key factors reported in the literature are categorized in this survey paper.

The fundamental microwave system used for wireless IBDs’ functionality comprises two main elements: a power supply and an antenna. Among these components, the antenna is the most significant one, as it governs the wireless functionalities and microwave energy distribution during the IBD’s activation time, as shown in Figure 2. While numerous studies have concentrated on quantifying the influence of thermo-electric parameters on treatment outcomes, there was minimal research conducted regarding the safety considerations of IBDs. As shown in Figure 3, the majority of the studies found in the literature that considered the IBDs’ thermal analysis and related safety assessments were related to thermal therapy-oriented applications. The limited availability of comprehensive information on this subject was the motivation behind presenting this survey paper. This article is intended to provide a comprehensive viewpoint regarding the impact of key factors such as the antenna design (i.e., structure and size), system settings (i.e., operating frequency, applied power, and treatment duration), testing environment (i.e., implantation depth and phantom type, size, and shape), and thermal model (i.e., blood perfusion, metabolic rate, and temperature-dependent properties) on the heat dissipation of IBDs reported in the literature during the activation of IBDs for different medical purposes, as it is summarized in Figure 4 and Table 1. We believe having access to prior information, a good understanding of previous studies, and powerful simulation methods equip clinical practitioners to safely integrate IBDs, accurately predict treatment outcomes, and minimize damage to healthy tissue, enhancing the overall reliability of the procedure.

## 3. Antenna Parameters

**Figure 5:**
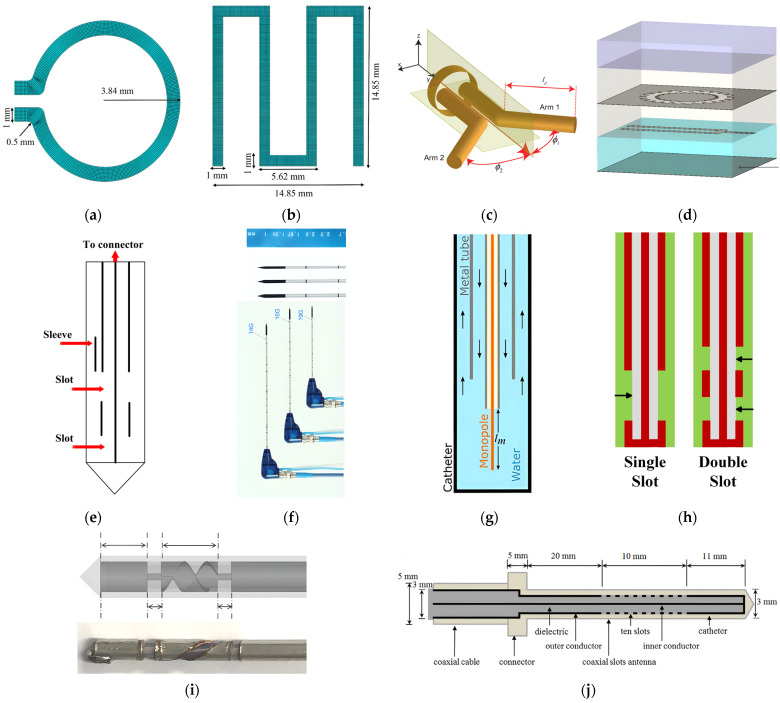
Examples of implanted antenna topologies: (**a**) loop [36]; (**b**) meandered line monopole [36]; (**c**) dipole [48]; (**d**) multilayer microstrip line-fed slot [50]; (**e**) sleeved monopole [56]; (**f**) coaxial [61]; (**g**) water-loaded monopole [50]; (**h**) single- and double-slotted coaxial [64]; (**i**) helical [75]; and (**j**) multiple slotted coaxial [69].

Numerous previous studies demonstrated encouraging outcomes of IBDs used for various treatment purposes. However, these studies often rely on several simplifying assumptions that do not represent the complex treatment environment [40]. These studies are often carried out utilizing either a two-dimensional (2D) model or ideal sources for the implanted antenna element, disregarding the actual radiation characteristics of a realistic implanted antenna that determine the actual direction and shape of traveling waves within the targeted body tissue [72,73]. Hence, the performance of optimized IBDs is closely linked to the antenna design, making its improvement an ongoing area of interest. An ideal implanted antenna should possess high-energy transmission efficiency for a long duration without damaging it, be noninvasive to patients, and maintain minimum heat dissipation levels to the healthy biological tissues, thus not causing hazardous effects. In addition to these requirements, in hyperthermia and ablation technologies, the antenna not only dictates the distribution of the produced heating but also influences the shape of the resulting ablation pattern (i.e., size and shape). Hence, it is important to consider the effect of the selected antenna’s structure, material, and size on the achieved thermal performance. The presented discussion focuses on the effect of changing these parameters on the antenna’s temperature profile during the IBD’s activation time and consequently, the heat dissipation inside the tissue and the comfort and safety of patients. On the other hand, when temperatures reach high levels, vaporization and carbonization take place, leading to changes in the complex permittivity of tissues, which affects the performance of the implanted antenna [74]. A summary of reported work in the literature on the thermal analysis of IBDs based on antenna-related parameters is presented in Table 1. Various implanted antenna modalities were proposed and extensively investigated via clinical, experimental, and computational studies [57]. For example, in the field of thermal therapy, several types of antennas such as slot, monopole, helical, and dipole coaxial-based antennas were widely employed for tumor ablation, aiming to non-surgically destroy cancerous tissues. These antennas are favored for their advantages, which include low manufacturing cost, compact size, simple design, and suitability for treatment purposes. Implanted antennas of different structures such as loop [36], meander [36], dipole [48], flexible multilayered [50], sleeved [56], coaxial [61], water-loaded monopole [50], single-slotted and double-slotted coaxial [64], helical [75], and multiple slotted coaxial [69] are shown in Figure 5.

The number of slots included in the structure of the implanted slot antenna, such as single- [39], double- [45], triple- [68], and multiple-slot [69], in addition to the spacing between the slots was found to have a significant impact on the obtained temperature profile of the implanted antenna. In [6], the influence of single-slot and double-slot antennas on the temperature distribution inside the liver was investigated. The highest temperature values were achieved using a single-slot antenna. In [45], it was reported that after 240 s of activation time, the maximum localized temperature values inside the breast tumor reached over 60° C for three different antenna configurations: a single-slot antenna, a double-slot antenna with a slot’s spacing of 4.05 mm, and a double-slot antenna with a slot’s spacing of 0.4 mm. In [57], the effects of coaxial slot antenna type (i.e., single-slot versus double-slot), slot positioning (i.e., slot-to-antenna tip distance), and slot size were investigated using parameters’ values that closely resemble realistic clinical scenarios. Findings indicated that double-slot antennas with slot-to-tip and slot-to-slot distances of approximately 10 mm were an optimal design choice. Such antennas can generate relatively uniform and localized temperature distributions near their tip to be used for MWA of liver tissue. Hence, they had a minimal impact on the healthy tissue. In [68], a new approach was proposed for the treatment of large-volume malignant liver tumors, utilizing multiple-point MWA with a novel triple-slot antenna. However, the damaged healthy tissue was not investigated, leaving unanswered safety concerns. In [69], a coaxial antenna with multiple preoperative, adjustable slots was proposed for conformal MWA inside liver tissue. Heating patterns can be modified by changing the number of or the spacing between antenna slots. This allows for lower temperature values close to the skin, potentially mitigating skin injuries. 

In [36], three antenna structures (i.e., dipole, loop, and meander monopole) operating at 7.14 GHz were modeled numerically to evaluate their thermal performance for use in the human body. In addition, these antennas were modeled using three different antenna materials (i.e., titanium, cobalt, and Macor). The meander antenna fabricated using titanium material demonstrated favorable characteristics in terms of reducing the antenna’s thermal impact on the biological tissue. The maximum localized temperature value was 37 °C. On the other hand, the loop antenna made of Macor material was the worst design choice, with a maximum localized temperature value of 1136.23 °C. In [56], the backward heating effect of a sleeved double-slot antenna was found to be the lowest when compared with single-slot, double-slot, and monopole antennas operating at 2.45 GHz. However, the highest power dissipation (i.e., 48.9 W) and temperature (i.e., 305.0 °C) values were recorded in the case of sleeved double-slot antenna when compared with other antennas. On the other hand, in [64], a comparative analysis of transient temperature due to the performance of different implanted antennas was carried out to determine the temperature field changes in terms of shape and size when implanted inside liver tissue. Single-slot, double-slot, monopole, and sleeved single-slot antennas were considered, and they all operated at 2.45 GHz. Results showed that the sleeved single-slot antenna was the most sensitive one, showing changes in the antenna reflection coefficient, hence affecting probe performance throughout implantation. In [48], an investigation was carried out to propose a new class of implanted antennas capable of generating heating patterns that possess either axially symmetric or asymmetric characteristics for thermal therapy purposes. This helps to efficiently deposit energy in the intended direction while reducing thermal impact on the surrounding biological tissue. The antenna system consists of a dipole that is fed by a balanced parallel-wire transmission line. The angle and orientation of the dipole arms were fine-tuned to regulate the intended heating pattern.

### Antenna Size

A rechargeable neuromodulation system was investigated in [17] using a layered body model. The proposed system consists of an implanted antenna (i.e., implanted inside fat tissue) and a wearable antenna (i.e., placed on skin tissue). A parametric study was carried out on the implant thickness and radius of the antenna to determine the optimum size of the proposed system, resulting in minimal heat dissipation. Higher temperature values were observed within the tissue as the antenna radius was increased, while the implant size was found to be the least influential design parameter. In [47], the performance of two implanted double-slot antenna, which was designed using semi-rigid coaxial cables with different diameters was compared. The thick antenna of 6.35 mm diameter produced less maximum localized temperature of 191.8 °C and reduced heating along the antenna shaft when compared to the smaller antenna size of 2.21 mm. The value of the temperature in the case of the smaller antenna was 227.1 °C. It is worth mentioning the input power was set to 50 W at 2.45 GHz in all cases. In [58], the performance of three implanted coaxial microwave antennas with outer diameters of 1.03 mm, 1.6 mm, and 2.0 mm implanted inside ex vivo porcine lung were evaluated. The temperature profile of the 1.03 mm diameter antenna was the same as those of the 1.6 mm and 2.0 mm diameter. This reduced the incidence of health complications when the antenna was implanted in lung tissue due to the smaller antenna size. The invasiveness of the wireless IBDs may be increased due to the use of rigid implanted antennas and their percutaneous insertion. An example where these limitations become pronounced is in the case of lung tumors. The placement of such tumors and their proximity to nearby organs such as the heart, blood vessels, and diaphragm may restrict the feasibility of using rigid implanted antennas [76]. Furthermore, reported studies have shown instances of pneumothorax resulting from the insertion of the rigid implanted antenna inside lung tumors [77,78]. As a result, rigid antennas are often replaced by flexible antennas to attain a less invasive implantation procedure with fewer complications. Temperature variations were observed when the implanted antenna was in a conformal state compared to a flat state. In [50], a compact conformal multilayer implanted antenna inside muscle tissue was investigated for different curvature radii of 50 mm, 100 mm, 200 mm, and 400 mm. Peak temperature in all considered cases was found to be aligned with the antenna’s central axis of radiation, causing hot spots in the superficial region of the tissue surface.

## 4. System Parameters

Frequencies primarily allocated for different wireless IBDs’ applications are summarized in Table 2. A higher operating frequency of IBDs results in smaller sizes of implanted antennas as compared to implanted antenna operating at a lower frequency, resulting in less invasive IBDs and opening up possibilities for creating more compact multielement devices that can provide heating and biotelemetry properties that cannot be achieved using single-element antennas [63]. However, with higher wireless IBDs’ operating frequency, the absorbed EM power tends to concentrate more in the vicinity of the skin tissue. This outcome is predictable because the penetration depth of waves decreases with an increase in frequency. Thus, the heating effect at the skin layer, the temperature rise, and safety requirements require careful examination of the effect of IBDs’ operating frequency [40]. In general, changes in frequency can alter the electrical conductivity and the permittivity of the biological material, while a higher applied power would result in increased input energy. Consequently, these variations have a significant influence on the quantity of heat being delivered to biological tissues [37]. To the best of the authors’ knowledge, only a limited number of previous studies that were related to thermal therapy applications have taken into consideration the effect of operational parameters of wireless IBDs, such as frequency, applied microwave power, and treatment time in the optimization of the relevant parameters. Some of these studies are summarized in Table 1. As an example, operating IBDs at higher frequencies offers several advantages. Firstly, it enables faster heating and reduces the thermoregulatory impact, as polar molecules rotate more rapidly. Additionally, the choice of operating frequency influences the ablation zone’s characteristics, determining the shape and size of the resulting heating pattern. While various antenna topologies were proposed for thermal therapy applications, most of the studies documented in the literature examined the treatment procedure at frequencies below 2.5 GHz, as summarized in Table 3. In particular, frequencies around 915 MHz and 2.45 GHz were widely used for thermal therapy purposes [63].

**Table 2:** Summary of frequency standards used for wireless IBDs [79].

Frequency Standard	Frequency Range
Inductive Implant	<100 kHz
Medical Device Radiocommunication (MedRadio)	(401–406) MHz
Medical Micropower Networks (MMNs)	(413–457) MHz
Medical Body Area Networks (MBANs)	(2.36–2.40) GHz
Ultra-Wideband Band (UWB)	(3.1–10.6) GHz
Industrial, Scientific, and Medical (ISM)	(433.1–434.8) MHz (868–868.6) MHz (902.8–928) MHz (2.4–2.5) GHz (5.715–5.875) GHz
Wireless Medical Telemetry Service Frequency (WMTS)	(608–614) MHz (1.395–1.4) GHz (1.427–1.429) GHz
Wi-Fi, Bluetooth, and Zigbee ^1^	(902–928) MHz (2.400–2.483) GHz (5.150–5.850) GHz (5.950–7.125) GHz

^1^ These frequency standards can be used for short-range digital modulation communication applicable to wireless IBDs.

**Table 3:** Development table of the frequencies and phantom types used in the thermal assessment of wireless IBDs.

	Frequency	Simulation Phantom	Measurement Phantom
[15]	2.45 GHz	Complex liver model	NA
[17]	NM	Layered model	NA
[19]	NM	NA ^2^	Porcine muscle
[31]	(3.5–4.5) GHz	Voxel model	NA
[33]	NM	Breast	NA
[35]	2.45 GHz	Homogeneous tissue	NA
[36]	7.14 GHz	NM ^1^	NA ^2^
[37]	0.1, 1, and 10 MHz	Tumor and healthy tissues	NA
[38]	2.45 GHz	Torso segment	NA
[39]	2.45 GHz	Liver	NA
[40]	(3–7) GHz	Breast	NA
[41]	1.55 GHz and 700 MHz	Simplified leg	NA
[42]	2.45 GHz	NM	Porcine liver
[43]	2.45 GHz	Bone	Porcine bone
[44]	(1.9–26) GHz	Porcine muscle and liver	Porcine muscle
[45]	2.45 GHz	Breast	Breast phantom
[46]	434, 650, 915, and 1150 MHz	Head model	NA
[47]	2.45 GHz	Liver	Bovine liver
[48]	10 GHz and 6.4 GHz	Muscle and egg white	Porcine muscle
[49]	915 MHz and 2.45 GHz	NA	Hepatic tumor
[50]	2.45 GHz	Muscle	NA
[51]	1.9, 6.0, 10, 14, and 18 GHz	Liver	Porcine liver
[52]	2.45 GHz	NA	Porcine liver
[53]	2.45 GHz	Lung	Porcine lung
[54]	2.45 GHz	Liver	Porcine liver
[55]	2.45 GHz and 6 GHz	Tumor	NA
[56]	2.45 GHz	Liver	Porcine liver
[58]	NM	NA	Egg yolk and porcine lung
[59]	2.45 GHz	Liver	Bovine liver
[60]	2.45 GHz and 5.8 GHz	Layered tissue model	Bovine liver and adrenal
[61]	1.9 GHz	Liver and egg white	Liver
[62]	2.45 GHz	Liver, lung, kidney, and bone	NA
[63]	10 GHz and 1.9 GHz	Liver	Bovine liver
[64]	2.45 GHz	Liver	NA
[66]	5 GHz	Homogeneous tissue	NA
[67]	2.45 GHz and 915 MHz	Liver	Porcine muscle
[68]	433 MHz	Liver	Porcine liver
[69]	2.45 GHz	Liver	NA

^1^ Not Mentioned (NM). ^2^ Not Applied (NA).

It is worth mentioning that, in most of the reported studies in the literature, analysis was carried out considering the combined impact of system parameters (i.e., frequency, applied power, and treatment time) on the treatment procedure and outcomes rather than an independent variation of system parameters. As an example, to achieve an ablation zone size of 5 cm diameter using a 915 MHz system that consists of three implanted antennas, approximately 45 W of input power was delivered via each of the antennas and required approximately 10 min of application time. In contrast, by using a 2.45 GHz system, the input power had to be set to 100 W using a single antenna and an application time of approximately 4 min to 6 min [80,81].

### 4.1. Operating Frequency

In [49], two commercial systems were employed to distinguish the differences between 915 MHz and 2.45 GHz systems utilized for hepatic tumor ablation. The comparison was based on the required amount of input power for lesion treatment. The 2.45 GHz system utilizes a single implanted antenna, while the 915 MHz system utilizes three implanted antennas [81]. According to the study findings, the 2.45 GHz system produced temperature distributions equivalent to the 915 MHz system. Nevertheless, it exhibited higher predictability and quicker achievement of the desired tissue heating, leading to fewer complications in the surrounding healthy tissues. 

### 4.2. Applied Power

In [65], numerical investigations showed that temperature distribution in liver tissue was significantly affected by the input power to the coaxial implanted antenna. It was observed that high input power resulted in increased temperature values (i.e., above 50 °C), which may destroy healthy tissues. On the other hand, with a power of 10 W, the tumor tissues could be effectively destroyed without causing harm to the adjacent healthy tissue. In [38], to explore the influence of input power on tissue damage depth and time, various power settings were investigated. For consistent and reproducible treatment experiments on a human male in the age range of approximately 25 to 30 years old, a torso segment was selected as the solution domain, encompassing liver, bone, muscle, fat, and skin tissues. As anticipated, the study results indicated that higher power levels led to increased localized tissue temperatures in a shorter period. For instance, when using an input power of 10 W within a 1 cm radius, it took more than twice as long to cause tissue damage to about 50% of the tissue cells compared to using 30 W (i.e., less than 100 s). 

### 4.3. Operating Frequency, Applied Power, and Treatment Time

In [37], the major parameters involved in EM heating inside healthy and tumor tissue types were investigated. Namely, parameters such as blood perfusion, metabolic heating rate, frequency, and input voltage were investigated. The findings showed that the applied voltage had the most significant impact on the maximum localized temperature, followed by the frequency of EM radiation, identified as the second significant factor. In fact, both input voltage and frequency were found to contribute to approximately 90% of the impact on the maximum attainable localized temperature. Interestingly, the interaction between all studied parameters was found to be insignificant. In [40], the impact of the frequency and input power on the focusing temperature during hyperthermia for breast cancer was investigated. The main goal of the study was to achieve the intended temperature at the targeted tissue after a given period of irradiation while preventing the occurrence of any elevated temperature zones within the healthy breast tissues. This study focused on the frequency band from 3 GHz to 7 GHz. This selection was made as a practical balance between the depth of penetration and the precision of effective treatment. Investigations suggest that depending on the breast density, the optimum frequency range for breast hyperthermia treatment is from 3.5 GHz to 4.5 GHz. In addition, shaping the temperature distribution inside targeted tissue can affect the treatment outcome, ultimately enhancing treatment efficacy. The heating pattern can be controlled and shaped by optimizing the excitation signal of the implanted antenna in terms of power amplitude and phase. In [44], floating-sleeve dipole antennas were designed at seven selected frequencies within the studied frequency range from 1.9 GHz to 26 GHz inside porcine muscle and liver tissues. The heating pattern of these antennas was evaluated as a function of the input power and applied for 5 min. The input power varied from 5 W to 40 W. The operating frequency and input power within the studied ranges substantially impact the heating pattern of designed antennas. Hence, trade-offs need to be considered among these variables to effectively control excessive tissue temperature rise and to minimize the risk of damaging the surrounding healthy tissue. In [53], MWA inside lung tissue was evaluated using a single-slot coaxial antenna operating at 2.45 GHz under different input power values of 20–60 W, with 10 W increments, and different treatment times of 2, 4, and 6 min. The findings of the study indicate that the temperature field increased gradually in ellipsoidal shape as the input power and application time were increased. In addition, the faster temperature rising was observed closer to the slot location. In [60], precise methods for creating short and spherical heating patterns to treat benign adrenal adenomas with volumes within the range of (0.5–4) cm^3^ (corresponding to diameters of (10–20) mm) were investigated. To achieve the desired heating patterns, adjustments of antenna operating frequency, input power, and application time were conducted. Specifically, the selected values for frequency were 2.45 GHz and 5.8 GHz, the input power was 30 W and 40 W, and the treatment time was 30 s, 60 s, and 90 s. The frequency of operation and treatment time were recognized as the main factors for regulating the length and width of the achieved heating pattern, respectively. In [63], despite the increase in the operating frequency by a factor of five, temperature profiles obtained at 10 GHz were comparable to results obtained at 1.9 GHz using different heating times of 5 min and 10 min. The applied power was kept at 42 W in all studied scenarios.

## 5. Testing Environment Parameters

Considering all the essential facts presented earlier, from a medical practitioner’s perspective, the influence of each factor during IBD activation for different treatment purposes is crucial and can affect the heat dissipation inside the biological tissue, consequently affecting the treatment outcome. It is important to identify the impact of each parameter utilized in the treatment procedure before performing and assessing actual clinical treatment [37]. Clinical trials were conducted to evaluate IBDs for different medical purposes. It is worth mentioning that the safety considerations in these clinical trials were limited to SAR standards without considering the actual heat dissipation due to IBD activation.

The reported studies in the literature were conclusive on the success of using both ex vivo and in vivo experiments in IBD evaluation and realization [66]. The numerical method can be used to define the temperature distribution of targeted biological tissue [23]. For example, finite element method (FEM), finite integration technique (FIT), finite difference time domain (FDTD), and method of moments (MoM) are commonly employed to discretize the partial differential equations in both time and spatial domains. Different simulation programs, including Abaqus (Dassault Systems), Icepak (ANSYS), COMSOL Multiphysics, FEKO (Altair), and CST Studio Suite (Dassault Systems) were equipped to offer comprehensive tools used to build and perform simulation applications related to antenna modeling, mimicking biological materials, mesh generation, selection of various physics components (i.e., EM and heat transfer modeling), and offering a good platform for evaluating results [56]. To confirm the feasibility of the proposed IBDs, investigating the robustness of the device against errors in tissue modeling is a crucial step in the design process. In addition, it ensures that heat dissipation in healthy biological tissues is kept at minimum levels [82]. Hence, conducting a closed-loop procedure that combines both EM and thermal simulations is important to ensure the absence of localized overheating inside the healthy tissues. A summary of used simulation programs in the literature, along with the co-simulation procedure is shown in Table 4. In addition, engineering simulation studies in parallel with clinical trials can help in defining the optimal parameters needed to design a treatment protocol specified for an individual patient, taking into consideration the patient’s physiological characteristics. A summary of reported work in the literature which have considered testing environment parameters in the IBDs’ heat dissipation evaluation, including implantation depth and testing phantom type, size, and shape is shown in Table 1.

### 5.1. Implantation Depth

In [43], temperatures in the range of 55 °C to 100 °C were reached inside porcine bone tissue using different levels of antenna insertion depths (i.e., 3.5 cm and 5 cm). The experimental results showed that the insertion of an implanted antenna equal to or less than 3.5 cm was not recommended. This is because such insertion depths modified the temperatures that could be reached inside bone tissue. The temperature distributions exhibited a concentration of heat in proximity to the antenna structure with a uniformly distributed thermal pattern. In [31], the EM effects of implanted antenna positioning inside the human voxel model on the surrounding biological tissues were investigated. It is worth mentioning that different placements of implanted antennas resulted in different implantation tissue types and different antenna orientations. Five positions were used inside the small intestine, while one position was used inside the colon tissue. An increase in the localized peak temperature of 0.438 °C above 37 °C used as the initial body temperature was recorded in the case of an antenna implanted inside the colon tissue. 

### 5.2. Phantom Type

In [40], various numerical breast models were employed for the hyperthermia treatment of breast cancer, as classified by the American College of Radiology [87]. These models represent a simple case of fatty tissue type in older women and a more complex case of younger women with very dense breast tissue. The employed models accurately replicate the physical form and structure of the human breast, consisting of eight distinct tissue types. These tissues include fat-1,2,3, glandular-1,2,3, skin, and muscle characterized by their specific levels of water content. It was found that to effectively focus the power on the targeted tumor inside a dense breast tissue, the optimal frequency is 4.2 GHz. This frequency is lower than the optimal value used for breasts of a fatty type because microwave signals experience higher attenuation when penetrating a dense breast. In both cases, the desired temperature is attained at the tumor location without causing any overheating in the surrounding healthy tissues. In [62], temperature distributions and damaged areas were evaluated for tumors located within the liver, lung, kidney, and bone tissues. System parameters were selected as 10 W of the input power, operating frequency of 2.45 GHz, and heating time of 600 s. Kidney tissue exhibited the highest value of temperature, while bone tissue recorded the lowest temperature. Importantly, in all examined tissue types, a tiny region of healthy tissue surrounding the tumor within bone tissue was overheated. As expected, the heat source is stronger in the vicinity of the implanted antenna, leading to high-temperature values. Conversely, far from the implanted antenna, the heat was weak, resulting in lower temperature values. In [33], the influence of three breast cancer-related parameters on the implanted antenna temperature distribution was investigated. These parameters were defined as (1) breast composition (i.e., extremely dense, heterogeneously dense, scattered fibro-glandular, and predominantly fatty), (2) tumor location affecting implanted antenna distance from the skin surface (i.e., 2.25 cm, 2.5 cm, 2.75 cm, and 3 cm), and (3) tumor size (i.e., 2 cm, 2.5 cm, 3 cm, and 3.5 cm). Among the studied parameters, breast composition had the most significant effect on the temperature pattern, followed by tumor location and size. More examples of phantom types used in the thermal analysis of IBDs, both in simulation and experimental evaluations are summarized in Table 3.

### 5.3. Phantom Size

In [45], the temperature distribution of different implanted antenna types inside breast phantoms of different sizes was evaluated. Spherical tumors with diameters of 1 cm and 1.5 cm were placed and tested inside the breast phantom. These scenarios were considered to simulate the use of MWA in early-stage breast cancer, which refers to stage 1 tumors with diameters less than 2 cm. Results demonstrated that when testing the tumor phantom with a 1 cm diameter, ablation temperatures (i.e., above 60 °C) were achieved in all tumor tissue areas using all tested antenna types. In contrast, when experimenting with a phantom tumor measuring 1.5 cm in diameter, the high-temperature profile extended over a 0.55 mm radius when using a single-slot antenna and 0.31 mm when employing a double-slot antenna. According to these results, in addition to the antenna geometry, the tumor dimensions modified the thermal pattern.

It is worth mentioning that small blood vessels located near the implanted antenna are susceptible to the risk of blood coagulation, which may potentially cause harm to the vessel walls. However, in the case of larger blood vessels, blood circulation tends to cool down and protect the large vessels from excessive heat damage. Reported studies in [88,89], which focused on small vessels with diameters less than 3 mm were found to be inadequate in meeting clinical requirements. In this case, further investigations are necessary for the temperature distribution of the implanted antenna in such scenarios. In [54], influences of different vessel diameters (i.e., 3 mm and 6 mm) and vessel-implanted antenna spacings (i.e., 17 mm and 5 mm) on the temperature distribution were analyzed. A 3D simulation model of liver tissue was established with dimensions of 50 mm and 100 mm, corresponding to the model radius and height, respectively. Ex vivo experiments using porcine liver tissues were constructed. Results showed that both spacing between the vessel and the antenna, in addition to the diameter of the larger vessel, affected the temperature distribution. It was found that spacing between the vessel and the antenna was the primary factor. 

### 5.4. Phantom Shape

In [55], the thermal profile of different-slot antennas was evaluated when inserted inside tumor tissue of three varying shapes (i.e., prolate, oblate, and spherical) and sizes. The study aimed to identify the most effective method for achieving the best treatment outcome while minimizing collateral damage. Results indicated that as the tumor size increased, a smaller size of cancerous cells was eliminated, and a higher level of side effects was noticed. Additionally, the tumor shape affected treatment efficiency. Elliptical tumors such as oblate and prolate were proved to be more challenging during treatment procedures compared to spherical tumors. Moreover, side effects were more noticeable in the scenario of oblate-shaped tumors than those obtained in the scenario of prolate-shaped tumors. In [15], a complex liver model consisting of blood, cancerous cells, and normal tissue was developed for the temperature distribution evaluation of an implanted antenna. The cancerous cells were modeled as three different sectional shapes (i.e., circular, horizontal oval, and vertical oval) with respect to the implanted antenna direction. Results showed that the shape of the tumor was one of the most important criteria for deciding the best antenna structure to use for achieving both effective cancerous cell death and normal cell safety. For tumors with an almost circular section, as well as those with an oval section that lies perpendicularly to the implanted antenna, a single-slot antenna would be the best instrument, whereas the dipole-tip antenna would be a good choice for the lowest backward heating, albeit with a lower temperature than that generated by the other antennas. For tumors with an oval section aligned with the antenna, the best choice would be the double-slot antenna.

## 6. Thermal Model Parameter 

Electromagnetic heating in biological tissue represents a bioelectromagnetic problem that involves the coupling of thermal models represented by bioheat equations and electromagnetic waves, both of which include variables that possess temperature sensitivity [64]. Various thermal models have been suggested in the existing literature [90,91] in order to calculate the temperature distribution due to IBD implantation within the biological tissue. To analyze heat transfer in tissues, the widely recognized Pennes’s bioheat equation [92] has been widely used. This equation is recognized for its relatively straightforward application and effectiveness in the majority of scenarios. It captures the impact of blood flow on the heat distribution in the biological tissue, including diffusion and cooling effects arising from the blood circulation represented as volumetrically distributed heat sinks or sources [57]. Hence, it permits the adjustment of the volumetric perfusion rate and the local arterial temperature, defined as the two main blood-related parameters, to modify the obtained results [17] described as follows:

(1) $$\rho c\frac{\partial T}{\partial t}=\nabla .k\nabla T+J.E-\rho_{bl}c_{bl}w_{bl}\left(T-T_{bl}\right)+Q_{m}$$

where ρ (kg/m^3^) is tissue density; c (J/kg.°C) is specific heat capacity; k (W/m.°C) is thermal conductivity; ***J*** (A/m^2^) is current density, ***E*** (V/m) is electric field intensity; $\rho_{bl}$ (kg/m^3^), $c_{bl}$ (J/kg.°C), $w_{bl}$ (1/s), and $T_{bl}$(°C) are blood mass density, blood specific heat, blood perfusion rate, and blood temperature, respectively; and $Q_{m}$ (W/m^3^) is metabolic heat generation.

Biological tissues generate heat via metabolic processes, and this heat production is an important factor in the thermal model. Metabolic heat generation depends on factors such as tissue type, activity level, and overall health. A reduction in the metabolic heat generation rate would increase the input energy. In addition, blood perfusion is a crucial parameter responsible for energy transfer via convection. If blood perfusion is elevated during IBD activation, it could potentially reduce the supplied energy from the implantation area, thereby hindering the creation of thermal lesions. Furthermore, in the case of thermal therapy, the blood flowing out of the treated region could cause unintended harm to the surrounding healthy tissue, making it challenging to achieve the desired treatment conditions [37]. Hence, blood perfusion acts as a heat sink that is particularly pronounced in highly perfused organs. One example is the liver, which is a blood-rich organ containing many small and large blood vessels [93]. It demonstrates a per-mass flow rate that is twice as large as that observed in other organs, such as the prostate and lung [94]. A summary of the research reported in the literature, which considered thermal model parameters in the IBDs’ heat dissipation evaluation, including blood perfusion, metabolic rate, and temperature-dependent tissue properties is presented in Table 1.

### 6.1. Blood Perfusion and Metabolic Rate

In [37], blood perfusion and metabolic heat generation parameters in healthy tissue and tumors were varied for three levels (i.e., high, medium, and low). The maximum achieved temperature was considered the response variable of the studied cases. Findings indicated that a reduction in the blood perfusion rate would result in a higher temperature value. This result holds significant importance for treatment planning purposes. A tumor is an intricate and irregular structure resulting from the uncontrolled proliferation of cells with distinct characteristics. Commonly, there is a major vein responsible for the primary blood supply to the tumor. If, before treatment, a reduced blood supply to the tumor is achieved via this major vein, it will be possible to achieve a higher temperature in a shorter period of time using the same treatment arrangement. The same conclusion was highlighted in [42] for the MWA of liver tissue. The effects of different blood perfusion rates (i.e., 0, 1, 1.5, and 3 kg/(m^3^.s)) on temperature distribution were systematically simulated and analyzed. The results suggested that if the blood perfusion is elevated, the achieved temperature can be decreased. In [44], two different in vivo environments were studied via simulations. The first one is representative of muscle tissue type with a blood perfusion coefficient [95] and metabolic heat generation [96] of 2647 (W/K/m^3^) and 706 (W/m^3^), respectively. The second one is representative of liver tissue type with a blood perfusion coefficient [95] and metabolic heat generation [94] of 71,232 (W/K/m^3^) and 10,931 (W/m^3^), respectively. In the case of high perfusion, it was shown that a lower level of the maximum temperatures was attained at 1.9 GHz, compared to higher studied frequencies. These findings align with the hypothesis proposed in [51], suggesting that heat diffusion has a crucial impact on the formation of thermal lesions during MWA treatment, particularly at frequencies above commonly employed 915 MHz and 2.45 GHz frequencies in MWA. The high rate of perfusion was demonstrated to significantly decrease the size of achievable thermal lesions by a factor of two to three when compared to non-perfused tissue. It is worth mentioning that egg white has been widely used in the literature ([48,58,61]) for non-perfused IBD experimentation because of many advantages such as simple operation, high consistency, low cost, and transparency facilitating real-time monitoring of the outcomes in three dimensions. In thermal therapy applications, it also aids in distinguishing the impacts of the antenna’s radiation from the distribution of shaft heating on the resultant heating profiles [38]. However, disregarding variations in attained temperatures caused by blood perfusion activities in ex vivo experiments could hinder the accurate assessment of the actual clinical output [66].

### 6.2. Temperature-Dependent Tissue Properties

**Figure 6:**
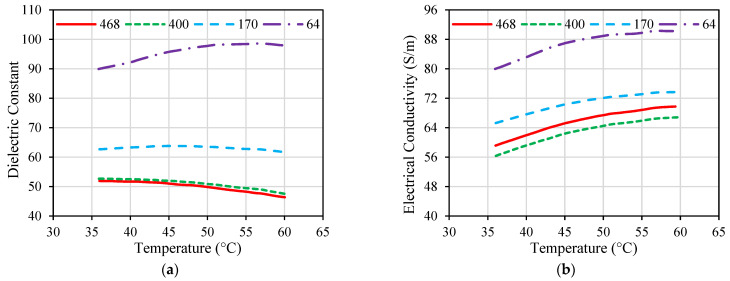
Dielectric properties of porcine liver tissue influenced by temperature at different frequencies given in MHz; (**a**) dielectric constant and (**b**) electrical conductivity. Data were recreated from [97].

**Figure 7:**
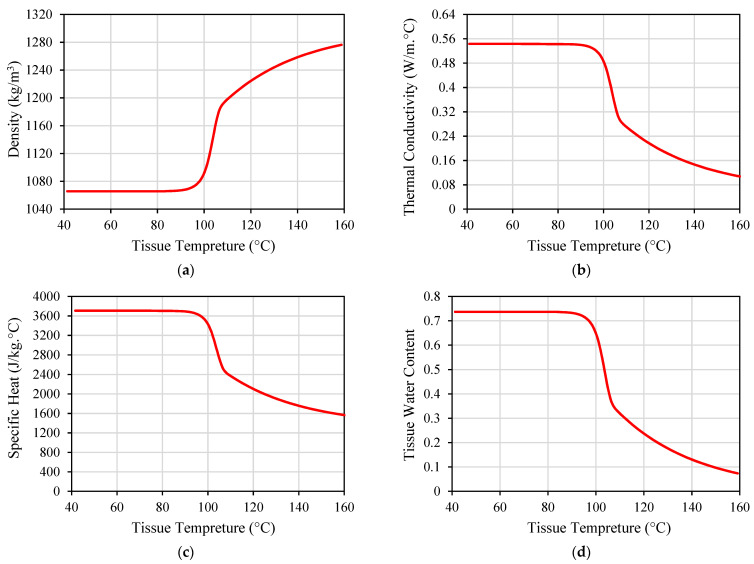
Thermal properties of porcine liver tissue influenced by temperature: (**a**) tissue density; (**b**) thermal conductivity; (**c**) specific heat; and (**d**) tissue water content. Data were recreated from [65].

While many implanted antennas were proposed in the literature, the majority of them were designed and evaluated based on the assumption of normal ambient tissue. Hence, they have not taken into account the changes in the tissue property that may occur [69]. Electrical and thermal parameters of biological material can change with the temperature variations, as shown in Figure 6 and Figure 7 for porcine liver tissue. When the temperature of liver tissue is increased from 37 °C to above 100 °C, a 5% change in tissue permittivity will take place [39]. In addition, the frequent changes in tissue properties can influence the temperature distribution in the proximity of the implanted antenna [67]. These variations are anticipated to shift the location of the absorbed microwave energy in the biological tissue. Hence, the accuracy of the numerical model employed to evaluate the microwave heating and hazardous effects due to IBD implantation is crucial for the success of the optimization approach based on the temperature factor. Temperature-dependent electrical and thermal parameters of the biological tissues should be considered during the design and evaluation phases of the IBDs to describe the actual variations of these parameters. There have been different mathematical models utilized in the literature to represent temperature-dependent electrical and thermal properties.

In [39], a linear approximation of liver tissue properties, including relative permittivity *ε_r_*, electrical conductivity *σ* (S/m), and thermal conductivity k (W/(m. °C)) was used to estimate the heating rate at 2.45 GHz within the temperature range of 37 °C to 100 °C, described as follows:

(2) $$\varepsilon_{r,liver}\left(T\right)=-0.0424\;T+47.043$$

(3) $$\sigma_{liver}\left(T\right)=-0.0004\;T+1.7381$$

(4) $$k\left(T\right)=0.0012^{T}+0.4692$$

In [42], empirical models that describe the relation between the tissue parameters and its temperature at 2.45 GHz are proposed to estimate the changes in the *ε_r_*, *σ*, *k*, and *c* as follows:

(5) $$\varepsilon_{r}\left(T\right)=45\;\left[1-\frac{1}{1+\exp\left(5.200-0.0519\;T\right)}\right]$$

(6) $$\sigma \left(T\right)=2.2\;\left[1-\frac{1}{1+\exp\left(5.324-0.0607\;T\right)}\right]$$

(7) $$k\left(T\right)=k_{at\;25\;℃}+0.00111\;(T-25)$$

(8) $$c\left(T\right)=\left\{\begin{array}{c} c_{\;at\;25\;℃}+K_{w\;}\;\left(T-25\right)\;,\;T\leq 70\;℃\\ c_{\;at\;70\;℃}-\frac{\propto }{\rho }\;\frac{\partial W\left(T\right)}{\partial T}\;,\;T>70\;℃ \end{array}\right\}$$

(9) $$W\left(T\right)=\left\{\begin{array}{c} 0.778\;\left(1-\exp\left(\frac{T-106}{3.420}\right)\right)\;,\;70\;℃\leq T<100\;℃\\ 7.053\;\left(1-0.0640\;T\right)\;,\;100\;℃\leq T<104\;℃\\ 0.778\;\left(1-\exp\left(\frac{T-80}{34.37}\right)\right)\;,\;104\;℃\leq T \end{array}\right\}$$

where $K_{w\;}$ represents the temperature coefficient (J/(kg. °C^2^)) and ∝ represents the latent heat constant (i.e., set to 2260 (kJ/kg)). It is worth mentioning that electrical properties (i.e., relative permittivity and conductivity) exhibit a decreasing trend as the temperature increases. This may cause a reduction in the material’s wave impedance that is proportional to the relation between the two properties. This is responsible for altering the matching and radiation properties of the implanted antenna throughout IBD activation [64,65,66,67,68,69,70,71,72,73,74,75,76,77,78,79,80,81,82,83,84,85,86,87,88,89,90,91,92,93,94,95,96,97,98]. 

In [59], two distinct dielectric property models were assessed at 2.45 GHz. Model 1 possessed a sharp decline in permittivity and conductivity at temperatures exceeding 60 °C [99]. Conversely, Model 2 utilized a comparable function but exhibited a smoother decline in permittivity as temperatures exceeded 100 °C, in contrast to Model 1 [100]. In [35,36,37,38,39,40,41,42,43,44,45,46,47,48,49,50,51,52,53,54,55,56,57,58,59,60,61,62,63,64,65,66], five thermo-electrical parameters, namely relative permittivity, electrical conductivity, heat capacity, thermal conductivity, and blood perfusion rate were divided into three levels based on the properties of liver, lung, breast, and kidney biological tissues. A homogenous biological tissue model was used at 2.45 GHz and 5 GHz. These studies are not focused on a specific tissue type but are aimed at understanding the impact of five critical factors on the heat dissipation of implanted coaxial single-slot antenna and helical antenna. 

It is worth mentioning that there are limitations on the availability of temperature-dependent properties data reported in the literature. In [60], temperature-dependent changes were only considered for heat capacity and thermal conductivity of liver tissue. Dielectric properties of liver tissue were assumed constant across all temperatures. This is because the temperature-dependent dielectric properties of liver tissue for temperatures exceeding 80 °C were only reported at 2.45 GHz. There is a lack of available data on liver temperature-dependent dielectric properties at 5.8 GHz. In addition, there are no published studies available that show the temperature dependency of adrenal tissue dielectric properties. In [62], temperature-dependent changes were considered only for relative permittivity and conductivity as described by (5) and (6) for liver, lung, kidney, and bone tissues modeled at 2.45 GHz. In [43], primarily due to the lack of available information regarding the thermal dependence of dielectric and thermal properties of bone tumors, the conducted studies focused only on healthy bone tissue in the temperature-dependent property models. In [69], liver tumor properties (i.e., density, thermal conductivity, and specific heat) were not accurately measured as a function of temperature variations [101]. As a result, these properties were assumed to be invariable throughout the study.

## 7. Conclusions 

The main goal of this research was to present the current state-of-the-art review of the latest advancements on the most effective parameters for the heat dissipation of IBDs reported in the literature during the activation of IBDs for different medical purposes. Critical parameters such as the antenna design (i.e., structure and size), system settings (i.e., operating frequency, heating power, and duration), testing environment (i.e., im-plantation depth and phantom type, size, and shape), and thermal model (i.e., blood perfusion, metabolic rate, and temperature-dependent properties) were comprehensively addressed and evaluated. This study can serve as a fundamental foundation to define the optimal parameters required to design a medical protocol that utilizes IBDs tailored to an individual patient, considering the specific physiological characteristics of the patient. It was demonstrated that the progress in computer technology and the availability of sophisticated computational tools enable strategic planning of personalized treatment and monitoring protocols. This approach aims to achieve maximum therapeutic benefits while minimizing adverse effects on the surrounding healthy tissues.

The provided and discussed collective characteristic observations showed that the effect of the system settings in terms of operating frequency, heating power, and treatment duration on the heat dissipation of IBDs were the most well-investigated parameters, spatially in the area of thermal therapies such as hyperthermia and MWA. On the other hand, in the context of other IBD medical applications, the heat dissipation effect needs to be further investigated, raising the necessity of carefully considering the selection of the most crucial parameters throughout the design cycle of IBDs to prioritize the parameters involved in the safety of IBDs. In fact, due to the lack of well-established standards that regulate heat dissipation due to IBD activation, the reported research work in the literature followed FCC regulations in terms of SAR standards to evaluate the IBD’s safety. The lack of available data on tissue temperature-dependent dielectric properties at different frequencies, such as liver, adrenal, and bone tumor tissues resulted in the assumption of constant tissue properties across all temperatures, hence affecting the accuracy of IBD thermal assessment. Finally, this research can be furthered by conducting a detailed and coupled EM and thermal analysis that considers the IBDs’ thermal effects. The study is advisable to consider the cumulative impact of the selected parameters on the heat dissipation and safety of future IBDs rather than an independent variation of the key design parameters. Hence, providing key solutions to the hazardous impact of IBDs that affect their functionality and treatment outcomes.

## Figures and Tables

**Table 1:** Development table of exciting thermal analysis studies of IBDs reported in the literature.

Ref.	Application	Antenna		System Parameters			Testing Environment				Thermal Model		
		Structure	Size	Frequency	Power	Time	Implantation Depth	Phantom Type	Phantom Size	Phantom Shape	Blood Perfusion	Metabolic Rate	Temperature Dependent
1_[36]	Other IBDs	X			X								
2_[19]	Other IBDs				X		X	X					
3_[17]	Other IBDs		X		X								
4_[37]	Hyperthermia			X	X			X			X	X	
5_[38]	Microwave Ablation	X			X								
6_[39]	Microwave Ablation	X											X
7_[40]	Hyperthermia	X		X	X			X					
8_[41]	Hyperthermia	X		X									
9_[42]	Microwave Ablation	X			X						X		X
10_[43]	Microwave Ablation					X	X						
11_[44]	Microwave Ablation		X	X	X			X			X	X	
12_[31]	Other IBDs				X		X						
13_[45]	Microwave Ablation	X							X				
14_[46]	Hyperthermia	X		X			X		X				
15_[47]	Hyperthermia	X	X			X							
16_[48]	Microwave Ablation	X		X				X					
17_[49]	Microwave Ablation			X									
18_[50]	Hyperthermia	X	X										
19_[51]	Microwave Ablation			X							X		
20_[52]	Microwave Ablation	X											
21_[53]	Microwave Ablation				X	X							
22_[54]	Microwave Ablation						X		X				X
23_[55]	Microwave Ablation	X		X	X				X	X			X
24_[56]	Microwave Ablation	X			X	X							
25_[57]	Microwave Ablation	X			X	X		X			X		
26_[58]	Microwave Ablation		X		X	X							
27_[59]	Microwave Ablation	X											X
28_[60]	Microwave Ablation			X	X	X					X		X
29_[61]	Microwave Ablation	X	X		X								
30_[62]	Microwave Ablation							X					X
31_[63]	Microwave Ablation			X		X					X		
32_[64]	Microwave Ablation	X											X
33_[65]	Microwave Ablation				X					X			X
34_[15]	Microwave Ablation	X								X			
35_[33]	Microwave Ablation						X	X	X				
36_[35]	Microwave Ablation				X						X		X
37_[66]	Microwave Ablation										X		X
38_[67]	Microwave Ablation	X		X							X		X
39_[68]	Microwave Ablation	X			X								
40_[69]	Microwave Ablation	X			X								X
41_[70]	Hyperthermia										X	X	X
42_[71]	Microwave Ablation								X				

**Table 4:** Some of the simulation programs reported in the literature used for the co-analysis of tissue heat and EM coupling modeling.

Software	References	Co-analysis Procedure
Abaqus	[36]	Based on the linear coupling between thermal and electrical elements. Heat generation from the EM analysis influences a heat transfer analysis, determining the temperature distribution. Simultaneously, the temperature distribution impacts the electromagnetic fields via temperature-dependent material properties [83].
ANSYS	[17,38]	Pennes’s equation is implemented utilizing commands of the functions of the parameters within the utility menu of the classical program. ANSYS-Thermal resolves the fundamental heat conduction equation ^1^ $\left(\rho c\frac{\partial T}{\partial t}=\nabla .k\nabla T\right)$, and then the related blood perfusion term ^2^ $\left(-\rho_{bl}c_{bl}w_{bl}\left(T-T_{bl}\right)\right)\;$ is integrated into the software. Ultimately, the additional function will be employed to generate heat across all regions of the model [84].
COMSOL	[39,42,43,45,46,47,53,54,55,59,60,61,62,66,67]	Mathematical models that involve coupled equations of EM wave propagation and bioheat equation are solved using 2D axisymmetric FEM [39]. The Helmholtz harmonic is employed to compute the EM energy deposition, while Pennes’s bioheat transfer equation is applied to address transient heat transfer within the tissue [85].
CST MWS	[40,41,44,51,63]	The results from the EM simulations, specifically the spatial distribution of the volumetric rate of microwave energy deposition in the tissue and the ohmic losses in the metals serve as the input (i.e., the heat source) for the thermal solver [86].

^1,2^ Details of equations are provided in Section 6.

## Data Availability

Data sharing not applicable.
